# A Comprehensive Review on the Current Vaccines and Their Efficacies to Combat SARS-CoV-2 Variants

**DOI:** 10.3390/vaccines10101655

**Published:** 2022-10-02

**Authors:** Ali A. Rabaan, Abbas Al Mutair, Khalid Hajissa, Amal H. Alfaraj, Jumana M. Al-Jishi, Mashael Alhajri, Sara Alwarthan, Shahab A. Alsuliman, Amal H. Al-Najjar, Ibrahim A. Al Zaydani, Ghadeer Hassan Al-Absi, Sana A. Alshaikh, Mohammed S. Alkathlan, Souad A. Almuthree, Abdulsalam Alawfi, Amer Alshengeti, Fatimah Z. Almubarak, Mohammed S. Qashgari, Areeg N. K. Abdalla, Saad Alhumaid

**Affiliations:** 1 Molecular Diagnostic Laboratory, Johns Hopkins Aramco Healthcare, Dhahran 31311, Saudi Arabia; 2 College of Medicine, Alfaisal University, Riyadh 11533, Saudi Arabia; 3 Department of Public Health and Nutrition, The University of Haripur, Haripur 22610, Pakistan; 4 Research Center, Almoosa Specialist Hospital, Al-Ahsa 36342, Saudi Arabia; 5 College of Nursing, Princess Norah Bint Abdulrahman University, Riyadh 11564, Saudi Arabia; 6 School of Nursing, Wollongong University, Wollongong, NSW 2522, Australia; 7 Nursing Department, Prince Sultan Military College of Health Sciences, Dhahran 33048, Saudi Arabia; 8 Department of Medical Microbiology & Parasitology, School of Medical Sciences, Universiti Sains Malaysia, Kubang Kerian 16150, Malaysia; 9 Pediatric Department, Abqaiq General Hospital, First Eastern Health Cluster, Abqaiq 33261, Saudi Arabia; 10 Internal Medicine Department, Qatif Central Hospital, Qatif 635342, Saudi Arabia; 11 Department of Internal Medicine, College of Medicine, Imam Abdulrahman Bin Faisal University, Dammam 34212, Saudi Arabia; 12 Infectious Disease Division, Department of Internal Medicine, Dammam Medical Complex, Dammam 32245, Saudi Arabia; 13 Drug & Poison Information Center, Pharmacy Department, Security Forces Hospital Program, Riyadh 3643, Saudi Arabia; 14 Department of Pediatric Infectious Diseases, Abha Maternity and Children Hospital, Abha 62526, Saudi Arabia; 15 Department of Pharmacy Practice, College of Pharmacy, Alfaisal University, Riyadh 325476, Saudi Arabia; 16 Diagnostic Virology Laboratory, Maternity and Children Hospital, Eastern Health Cluster, Dammam 32253, Saudi Arabia; 17 Infectious Diseases Department, King Fahad Specialist Hospital, Buraydah 52382, Saudi Arabia; 18 Department of Infectious Disease, King Abdullah Medical City, Makkah 43442, Saudi Arabia; 19 Department of Pediatrics, College of Medicine, Taibah University, Al-Madinah 41491, Saudi Arabia; 20 Department of Infection Prevention and Control, Prince Mohammad Bin Abdulaziz Hospital, National Guard Health Affairs, Al-Madinah 41491, Saudi Arabia; 21 Department of Family Medicine, Family Medicine Academy, Dammam 36365, Saudi Arabia; 22 Communicable Diseases Prevention Department, Saudi Public Health Authority, Riyadh 13354, Saudi Arabia; 23 Department of Intensive Care Unit, Saudi German Hospital, Dammam 32313, Saudi Arabia; 24 Administration of Pharmaceutical Care, Al-Ahsa Health Cluster, Ministry of Health, Al-Ahsa 31982, Saudi Arabia

**Keywords:** SARS-CoV-2, viral variants, vaccine, immunity, mutations

## Abstract

Since the first case of Coronavirus disease (COVID-19) caused by severe acute respiratory syndrome coronavirus 2 (SARS-CoV-2) in 2019, SARS-CoV-2 infection has affected many individuals worldwide. Eventually, some highly infectious mutants—caused by frequent genetic recombination—have been reported for SARS-CoV-2 that can potentially escape from the immune responses and induce long-term immunity, linked with a high mortality rate. In addition, several reports stated that vaccines designed for the SARS-CoV-2 wild-type variant have mixed responses against the variants of concern (VOCs) and variants of interest (VOIs) in the human population. These results advocate the designing and development of a panvaccine with the potential to neutralize all the possible emerging variants of SARS-CoV-2. In this context, recent discoveries suggest the design of SARS-CoV-2 panvaccines using nanotechnology, siRNA, antibodies or CRISPR-Cas platforms. Thereof, the present comprehensive review summarizes the current vaccine design approaches against SARS-CoV-2 infection, the role of genetic mutations in the emergence of new viral variants, the efficacy of existing vaccines in limiting the infection of emerging SARS-CoV-2 variants, and efforts or challenges in designing SARS panvaccines.

## 1. Introduction

According to the WHO report, around 572,239,451 cases of COVID-19 have been confirmed globally, with 63,904,401 deaths confirmed through 29 July 2022. The maximum confirmed 240,211,364 cases of COVID-19 were detected in Europe, while the lowest 92,091,133 cases were reported in Africa. In addition, in terms of death caused by SARS-CoV-2, the United States of America (USA) displayed 1,020,405 death reports, while the lowest death rate was noted in Africa. A summary of the reported deaths caused by COVID-19 worldwide is mentioned in Table 1. It is also important to mention that about 12,248,795,623 doses of different vaccines designed against SARS-CoV-2 have been administered worldwide, and the number of vaccinations in percent per country (top 10 countries in the world) is also cited in Table 2.

Initially, multiple vaccine strategies and candidates were reported soon after the pandemic caused by SARS-CoV-2 but proved unsuccessful in the control and management of COVID-19. Experts observed that the emergence of new viral variants caused by the acquisition of genetic mutations in SARS-CoV-2 in some parts of the world and then rapid transmission across the continents essentially contributed to the progression of COVID-19. In addition, the available vaccines showed varying efficiencies against the emerging SARS-CoV-2 mutants depending on the population and variants. This highlighted the need to develop a broad-spectrum vaccine that can neutralize all the existing and future variants of SARS-CoV-2. Herein, we comprehensively describe the vaccine development strategies against SARS-CoV-2 and different vaccine candidates. We also highlight the efficiency of existing vaccine candidates against the SARS-CoV-2 variants. Finally, we comprehensively describe the recent attempts to develop a broad-spectrum vaccine formulation that can neutralize the existing variants of SARS-CoV-2.

## 2. Methods

In this review, the PubMed database was used to search and analyze the reported publications pertinent to the present review’s title related to vaccines associated with SARS-CoV-2. The Preferred Reporting Items for Systematic Review and Meta-Analysis (PRISMA) were used in the search methodology. The keyword string created for the search method, composed of (((COVID[Title] OR SARS[title] OR COV[title]) AND (vaccine[Title/Abstract])) AND (Journal Article[Publication Type] OR Review [Publication Type])) was also included for the literature collection from the PubChem database. Initially, the above keywords were searched using the default publication type, which consisted of a journal article and review under the title section, to improve the relevancy. As a result, a total of 17,102 articles were obtained. Consequently, a variety of filter criteria were employed to cut down the number of articles and identify only those that were more pertinent to the aim of the present review. For instance, in the first filter, the publication date was used with the most recent five years’ publications, which yielded 16,838 publications. Following the year-long criteria, a clinical trial criterion was implemented. As vaccine development and testing can be evaluated using clinical trials, clinical trial articles were also considered in this review. A total of 258 clinical trial articles were available on the subject of interest, while other relevant observational studies reported in 280 articles were also considered. Recently, therapeutic solutions for COVID-19 have been reviewed exhaustively, making the review articles, systematic reviews, and meta-analysis articles crucial to be considered in this study. This produced a total of 2975 articles, whereas 108 were only meta-analyses. These articles were further processed for common publications by using PMID were removed. Unique articles were used in searching for keywords [variant OR effect OR effectiveness OR feasibility OR action OR risk OR efficacy OR factors OR features OR efficacious] in the abstract. This produced 129 as the most relevant articles that were considered in drafting this review.

## 3. Vaccine Development Strategies against SARS-CoV-2

### 3.1. Inactivated Vaccine

In vitro culturing of viruses and their chemical inactivation is one method for developing vaccines. These vaccines may deliver consistently expressed, conformationally native antigenic epitopes. The firms with the most advanced vaccines are Sinopharm and Sinovac. These products have undergone evaluation in Phase 3 studies and received worldwide use authorizations [1,2,3,4].

### 3.2. Protein Subunit Vaccine

Delivering the recombinant viral spike protein through the cellular-based systems that mediate the protein production is another strategy for vaccine production. This strategy can provide in vivo protection for vaccinated animals, but it theoretically runs the danger of inducing a polarized immune reaction that can be managed using different adjuvants [5]. Recently, Novavax published the results of its last phase of clinical testing in the UK, showing vaccination effectiveness against COVID-19 of 89% while employing the saponin-based Matrix-M adjuvant. Though none are approved for use, over 60% of vaccines presently under study employ a protein subunit method [1,2,3,4].

### 3.3. Vaccines on Viral Vector

In viral vector-based vaccinations, replication-deficient viruses express the gene sequence of a target antigen inside the host cells. Adenoviruses incapable of replicating themselves have been created for the HIV, TB, ebola, and malaria viruses [6]. The effectiveness of this vaccination strategy has varied, frequently being constrained by previously existing immunity to the adenovirus vector [7]. The vaccines employing the Adenovirus serotype 26 vector vaccine (Ad26.CoV2.S; Johnson and Johnson) have shown early success using adenoviruses with low levels of innate immunity in the US and Europe (ChAdOx; AstraZeneca). Both have varying degrees of effectiveness in avoiding clinical disease, especially disease brought on by SARS-CoV-2 variants, but are effective in minimizing COVID-19-related hospitalization and mortality [1,2,3,4].

#### 3.3.1. Replication Deficient Viral Vectors

A sizable category of vaccines in development are replication-incompetent vectors. These vaccines employ different viruses whose genomes are modified to produce the structural protein of SARS-CoV-2 and have portions of their genome deleted to prevent them from replicating in vivo. Many viruses, including human influenza and parainfluenza viruses, modified vaccinia Ankara (MVA), adenoviruses, and the Sendai virus, are also used [8,9,10,11,12,13,14]. The adenoviruses (AdV)-based vectors account for most of these strategies. Most of these vectors are administered via intramuscular injections that penetrate deep inside the tissues to produce viral spike proteins. This strategy does not involve working with live SARS-CoV-2 for vaccine manufacture. Additionally, there is extensive expertise in making more significant quantities of these vectors. It is a drawback that some of these vectors are impacted by pre-existing vector immunity and are partially neutralized by it [10]. Using virus-based vectors, which are not familiar to humans [9], generated from animal viruses [11], or viruses that do not produce much protection on their own are ways to get around such immune responses (for example, adeno-associated viruses). Additionally, when using prime-boost treatments, vector immunity might be troublesome; however, it can be minimized by priming two viral vector-based vaccines. The data from clinical trials of several replication-deficient vector vaccine applicants against SARS-CoV-2 has advanced significantly. The outcomes from human clinical trials are disclosed [9,10,11,12]. In addition, a vaccine candidate (Ad5/Ad26) [15] is in Phase III clinical trials, and another from ReiThera (gorilla AdV) is in Phase I trials (https://www.who.int/publications/m/item/draft-landscape-of-COVID-19-candidate-vaccines; 1–4) (accessed on 7 August 2022).

#### 3.3.2. Replication Competent Viral Vectors

The gene encoding the SARS-CoV-2 spike protein can be introduced into the weakened or vaccine strains of viruses to produce replication-competent vectors. Animal viruses that do not multiply well and do not infect humans are occasionally employed. This method may induce a more robust immune response due to the vector’s capacity to propagate to some degree inside the vaccine recipient and the frequent triggering of a strong innate immune response. Several of these vectors may also be administered directly to the mucosal lining, leading to immune responses inside the mucosa. Two such vectors are being tested in clinical trials (Phase I), i.e., one measles strain and another influenza strain. However, several others are now being developed, including those employing the vesicular stomatitis virus, i.e., VSV [16], horse pox, and Newcastle disease virus, i.e., NDV [17,18]. Interest in NDV-based vectors stems from the virus multiplying to produce high titers in eggs. These vectors can also be modified using the already-existing framework being used for influenza virus vaccines. They are probably safe enough just to deliver intra-nasally, unlike measles and the VSV vectors, which might produce mucosal immunity [1,2,3,4].

### 3.4. DNA Vaccines

A plasmid carrying the DNA encoding for virus proteins can be multiplied to obtain enormous quantities in bacteria. This forms the basis for DNA vaccines. These plasmids usually include the spike protein gene and mammalian expression promoters, which are generated in the recipient of the vaccination. The capacity to produce in vast quantities in *E. coli* and the high level of plasmid DNA stability are two of these technologies’ most significant advantages. Furthermore, DNA vaccines frequently exhibit limited immunogenicity, necessitating the use of delivery mechanisms to make them effective. This necessity constrains the utilization of delivery tools such as electroporation. Phase I/II clinical studies for four DNA vaccine candidates against COVID-19 are ongoing (https://www.who.int/publications/m/item/draft-landscape-of-COVID-19-candidate-vaccines; 1–4) (accessed on 7 August 2022).

### 3.5. mRNA Vaccines

The development of vaccines against various infections might be significantly enhanced by recent developments that use mRNA for vaccine delivery. Lipid nanoparticles are employed in these vaccines to safeguard the mRNA (encoding the virus S protein) within the cellular milieu. Inside the host cells, translation occurs to produce the target protein, the S protein, thus triggering a well-coordinated immune response. In clinical studies, mRNA-based vaccines created by Pfizer-BioNTech and Moderna showed over 90% effectiveness against the clinical illness caused by COVID-19. This strategy has several benefits, including quick vaccine production (weeks) and the capacity to elicit a T_H1_ or T_H2_ reaction. Studies are being conducted to evaluate the effectiveness of the presently approved vaccine candidates in protecting against the COVID-19 variants in children and determine the efficiency of booster shots that include the variants’ mRNA [1,2,3,4].

These vaccine approaches are relatively similar to other types of viruses known to possess a faster mutation rate. For instance, in the case of the Influenza, the four major strategies used for conventional vaccine approaches include inactivated, live attenuated, recombinant, and cell-based vaccines. Inactivated vaccines, in particular, can induce a strong humoral response with a predominance of IgG than IgA [19]. The live attenuated vaccine strains are generated by incorporating the significant antigen-encoding genes HA and NA from the genome of seasonal or circulating variants into the backbone of a heat-sensitive weakened influenza virus [20]. These modified influenza viruses generate a humoral immune response in the upper respiratory tract and also a cell-mediated immune response [19]. The recombinant vaccine, such as Flublok, contains the HA antigens from seasonal influenza strains produced and purified in insect cells [21]. Flucelavax is an inactivated vaccine that is produced by growing the virus in mammalian cells [22]. The inactivated vaccine remains the popular choice for the influenza virus, but the seasonal variants have to be used for the vaccine preparation, which otherwise may lead to inadequate protection [23]. In addition, the use of subunit vaccines such as DNA or mRNA-based vaccines has been shown to boost the immune response to other vaccine candidates, thus contributing to vaccine priming [24]. Recently, influenza vaccines have been modified to specifically activate a specific set of immune cells within the host. These T-cell-targeted vaccine candidates mainly consist of highly conserved epitopes with the potential to stimulate cytotoxic T cells. For example, various internal and surface proteins in the zoonotic influenza virus contain epitopes that are reactive against T cells. A multimeric-001 vaccine is another example. It has nine epitopes that are conserved in both the A and B strains of the influenza virus [25].

**Figure 1:**
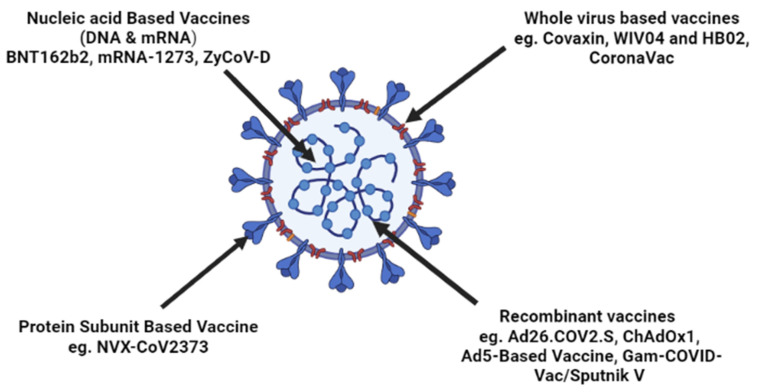
Strategies illustrating the type of vaccine implemented in controlling the SARS-CoV-2 spread.

Various vaccine strategies against SARS-CoV-2, along with their examples, have been summarized in Figure 1.

## 4. Vaccines Currently in Usage

### 4.1. BNT162b2

This vaccine is produced by Pfizer-BioNTech and contains the mRNA encoding the viral S protein encapsulated inside the lipid nanoparticle and then administered to produce a full-length spike protein. In the initial months following BNT162b2 immunization, there is a significantly lower incidence of symptomatic and severe COVID-19, according to randomized studies in kids and adults. In big placebo-controlled trials, the two-dose primary sequence of the vaccine was a 95% confidence interval [CI] with 90.3–97.6 effectivity at stopping symptomatic COVID-19 in adults age 16 or more [26] (https://www.fda.gov/media/153714), (accessed on 19 September 2022), 100% (95% confidence interval [CI] 75.3–100) effective at trying to prevent COVID-19 in adults aged 12–15 years [27], and 91% effective at preventing symptomatic COVID-19 in children aged 5 to 11 years (https://www.fda.gov/media/153714), (accessed on 19 September 2022). Vaccine effectiveness was 91.7% in persons under 65 with obesity or other associated complications (95% CI 44.2–99.8). Following a longer period of time, vaccine effectiveness stayed high but somewhat fell to 90% at 2 to 4 months and 84% at 4 to 6 months [27]. Among approximately 50,000 study participants over six months, just 1 of 30 serious infections (i.e., involving hypoxia, organ malfunction, or critical illness) occurred in a vaccine recipient. The effectiveness of the vaccination was 80.4 % (95% CI 14.1–96.7) in a short study involving kids aged six months to four years, although the estimation was unclear due to the small number of cases (https://www.fda.gov/media/159195) (accessed on 7 August 2022). Two participants—both vaccination recipients who also had coinfections with other respiratory viruses—were seen in the ER or hospitalized due to COVID-19.

The trial results in adults and adolescents are supported by observational data from several nations after nationwide roll-outs of BNT162b2 [28,29,30,31,32,33,34,35,36,37,38,39,40,41,42,43]. Administration of BNT162b2 has greatly influenced the efficacy of avoiding admission to an intensive care unit, hospitalization, and mortality in adults and adolescents. Even though vaccination still significantly lowers COVID-19-related hospitalizations in this age group, even with the contingency, some observational evidence, though not all, suggests that vaccine usefulness in children between 5 and 11 years may be lower than that among older adolescents. However, it is uncertain how much of that difference is linked to decreased vaccine effectiveness against the Omicron variant, which predominated shortly after the emergence of vaccines for younger kids [33,44,45,46,47,48,49,50]. The efficacy of vaccines declines with time and may be reduced in preventing infection with specific SARS-CoV-2 variations, while protection against severe illness brought on by variants continues to be significant. These effectiveness results are in line with data from immunogenicity studies, which showed BNT162b2 to elicit potent binding and to neutralize antibody responses with considerable age-related variability [26,51] (https://www.fda.gov/media/153714) (accessed on 7 August 2022). People over the age of 65 had responses that were typically lower than those of younger participants but were on par with or higher than titers in convalescent plasma. (https://www.fda.gov/media/159195,https://www.who.int/publications/m/item/draft-landscape-of-COVID-19-candidate-vaccines) (accessed on 7 August 2022) Children under five needed three shots (at a lower dose) to get the same level of neutralizing antibodies as older people who only needed two doses. Following BNT162b2 vaccination in adults, neutralizing antibody titers decreased over time; in one trial, men, those over 65, and people with impaired immune systems saw more significant losses in neutralizing titers for six months [52]. In comparison to activity against previously circulating strains, neutralizing activity is lower against the delta variation (B.1.617.2) [53,54,55] and still lower against the omicron variant (B.1.1.529). According to preliminary findings published in a manufacturer’s news release, three doses of a low-dose BNT162b2 formulation caused antibody responses in children aged six months to five years, compared to those reported in young adults after two normal doses (https://www.pfizer.com/news/press-release/press-release, (accessed on 7 August 2022) [1,2,3,4].

The virus-neutralizing antibodies are observed one month post-vaccination. The waning of antibody titers is delayed even after six months of vaccination. However, comparatively lesser activation of SARS-CoV-2 spike protein-specific CD^4+^ T cells is observed [56].

The side effects are relatively more common after the second vaccination dose than the first one. From 14 clinical studies with data available on the PubMed database, conducted on approximately 10,000 patients, Dighriri et al., analyzed the relative percentage of various side effects post-vaccination with BNT162b2. It was observed that 77% of people felt local pain at the site of injection, 43% experienced fatigue, 39% shared muscular pain, 33% had swelling at the injection site, and an equal percentage of people experienced a headache. The less common symptoms included joint pain (25%), chills (18%), fever (18%), itching (9%), lymphadenopathy (7%), nausea (7%) and diarrhea (6%) [57].

### 4.2. mRNA-1273

The first vaccine to be created against SARS-CoV-2 was this messenger RNA (mRNA) vaccine created by Moderna. To make the total spike protein, the vaccine uses mRNA that is given in lipid-based nanoparticles.

During the initial months of mRNA-1273 immunization, randomized clinical trials in adults displayed a considerably lower incidence of symptomatic and severe infection by SARS-CoV-2. The effectiveness of the two-dose main vaccine series in controlling symptomatic COVID-19 conditions among individuals 18 years of age and older was 94.1% (95% CI 89.3–96.8) in a significant placebo-controlled experiment [58]. The effectiveness of the vaccination was 86.4% in people under 65. (95% CI 61.4–95.5). The efficacy of the vaccine was 93.2% for symptomatic infection after a median follow-up of 5.2 months (9.6 versus 136.6 cases per 100 person-years with the placebo) and 98.2% for severe disease (i.e., with hypoxia, organ dysfunction, or acute illness; 2 versus 106 cases with the placebo) [59]. Although the estimates of impact were uncertain due to a few cases, limited trial data also suggest that vaccination efficacy against symptomatic COVID-19 is higher in children between 12–17 years (100%, 95% CI 28.9-non evaluable) and aged 6 to 11 years (69%, 95% CI −131.4–95.8) (https://www.fda.gov/media/159189) (accessed on 7 August 2022). Even though this estimation is primarily consistent with observational evidence on vaccination effectiveness against omicron in adults, efficacy was lower (41.5%, 95% CI 23.8–55) in a study of younger children that was conducted when the omicron variant was circulating. None of the trials, including those in children, had any significant cases of COVID-19.

The trial results in adults are further supported by observational data assessing vaccination efficacy [28,40,41,43,60,61]. Mainly, mRNA-1273 has been linked to a 90% or greater vaccination efficiency in avoiding emergency room visits, hospital admissions, admission to critical care units, and fatalities due to COVID-19 (1–4, https://www.who.int/publications/m/item/draft-landscape-of-COVID-19-candidate-vaccines) (accessed on 7 August 2022).

The efficacy of vaccines declines with time and may be reduced in preventing infection with specific SARS-CoV-2 variants, while protection against severe illness brought on by variants continues to be significant. These effectiveness findings are in line with data from immunogenicity trials that showed that people of all ages had strong binding and neutralizing antibody responses to mRNA-1273 [62,63]. Immunogenicity is on par with or greater than that observed in young adults in children or adolescents ages 6 months to 5 years (with a quarter-dose), 6 to 11 years (with a half-dose), and 12 to 17 years (with a regular dosage) (https://www.fda.gov/media/159189) (accessed on 7 August 2022) [64,65]. Antibody titers gradually decrease after six months, although they continue to be high, and neutralizing activity endures [66]. Compared to BNT162b2, vaccination with mRNA-1273 results in greater antibody titers following the second dose [67,68]. In comparison to activity against previously circulating strains, neutralizing activity is lower against delta [54] and significantly lower against Omicron variants. In a clinical trial involving various doses of mRNA-1273 vaccine, the lower doses of mRNA-1273 vaccine were observed to induce long-lived memory T cells, further enhanced by their cross-reactivity. In addition, the titer of neutralizing antibodies was maintained in 88–100% of cases for a minimum of 6 months after the second vaccine dose [69]. In a recent study which involved the comparison of the induction of humoral and cellular responses after the administration of different vaccines, similar findings were observed [70].

The Phase 3 clinical trial results showed immediate reactions at the injection site in 84% of the patients following the first dose of the vaccine, while 0.8% experienced delayed reactions at the site of injection. The other symptoms included weakness, chills, headache, nausea, sweating, and muscle spasms [71].

### 4.3. NVX-CoV2373

SARS-CoV-2 spike glycoproteins, along with a strong Matrix (M1) adjuvant, make up this recombinant protein subunit vaccine produced by Novavax. NVX-CoV2373 exhibited a 90.4% (95% CI 82.9–94.6) effectiveness in avoiding symptomatic COVID-19 in seronegative people between 18 and 84 years in a phase III efficacy trial conducted in the United States and Mexico [72]. The four severe instances happened in the group receiving placebos. Due to the modest number of illnesses in this category, the estimate of vaccination efficiency was lower but less clear among individuals 65 years of age or older (78.6%, 95% CI −16.64 to 96). A phase III study in the United Kingdom [1,2,3,4,73] revealed similar vaccination effectiveness (89.7%, 95% CI 80.2–94.6).

In the case of NVX-CoV2373, the neutralizing antibody titer peaked between 3.5 and 6 months after vaccination and was comparable to the mRNA vaccines, BNT162b2 and mRNA-1273, but higher than Ad26.COV2.S. The CD^4+^ T cells specific to the viral spike protein as well as the CD^8+^ memory T cells were only detected in 10–50% of the recipients [70].

The most common side effects include headache, fatigue, and malaise and persist for 2–3 days, while the systemic effects include myocarditis and pericarditis [73].

### 4.4. Ad26.COV2.S

This vaccine’s foundation is an adenovirus 26 vector that cannot replicate itself and encodes a stabilized spike protein. Janssen/Johnson and Johnson develop it. Randomized studies in adults have shown that after getting the Ad26.COV2.S vaccine, the chances of getting COVID-19 symptoms and a severe case are much lower. At a median two-month follow-up, a placebo-controlled trial showed that adults aged 18 and older were 66.9% effective (95% CI 59.0–73.4) at trying to prevent moderately severe COVID-19 (which also included patients with pneumonia, dyspnea, tachypnea, or at least 2 symptoms of COVID-19). After 14 and 28 days post-vaccination, the vaccine’s effectiveness against severe/critical infections (i.e., those accompanied by hypoxia, organ failure, or critical illness) trended higher at 78–85%. Effectiveness estimates were 56.3 % (95% CI 51.3–60.8) for at least moderate COVID-19 and 74.6% (95% CI 64.1–82.1) for severe/critical COVID-19 after a median of four months of follow-up [74]. Press reports claimed that a two-dose series had superior success rates (75 and 100% against symptomatic and severe COVID-19), but published study information is required to evaluate these claims rigorously [1,2,3,4,65].

A single dose of Ad26.COV2.S has been linked to vaccination effectiveness of 67–75% against COVID-19-related emergency medical care and hospitalization and 83 percent against COVID-19-related death [28,75,76]. Observational evidence analyzing vaccine effectiveness broadly confirms the trial results. These effectiveness results are in line with findings from immunogenicity studies that showed post-vaccination binding and neutralizing antibody responses that coincided with but were somewhat below those in convalescent plasma [74,77]. In contrast, neutralizing antibody levels after mRNA vaccination decrease over time (though remain higher than after Ad26.COV2.S). [78]. These neutralizing responses are relatively constant over eight months with both one- and two-dose regimens [79]. In addition, there is still neutralizing activity against the Delta (B.1.617.2) variation, but it is not as strong as it was against the Omicron (B.1.1.529) strains that were circulating before (https://www.who.int/publications/m/item/draft-landscape-of-COVID-19-candidate-vaccines) (accessed on 7 August 2022). In a comparative study, 64% of the Ad26.COV2.S recipients showed detectable neutralizing antibodies after 2 months, less than the mRNA vaccines. The presence of spike protein-specific CD^4+^ T cells was stable for 6 months post-vaccination. The peak levels of these antigen-specific T cells were still lower than the mRNA vaccines [70].

The side effects include breathing difficulties, chest and abdominal pain, thrombocytopenia and myocarditis [80].

### 4.5. ChAdOx1 nCoV-19/AZD1222

This vaccine was developed based on an adenovirus vector from a chimpanzee that produces the spike protein. Two dosages are injected intramuscularly. The two doses should be administered eight to twelve weeks apart, according to the World Health Organization (WHO’s recommendations for the use of AZD1222).

Randomized adult studies show that the risk of COVID-19 with symptoms is much lower in the first few months after vaccination. The vaccine’s effectiveness in avoiding COVID-19 after a follow-up of two months in large placebo-controlled studies ranged from 70–76% (95% CI 54.8–80.6) after 14 days of receiving the second dose [81,82]. According to further analysis of this experiment, receiving the second dose at 12 weeks or later was linked to greater vaccination effectiveness than receiving it at less than six weeks (81 versus 55%) [81]. These results provide evidence in favor of a 12-week delay between the first and second doses.

The trial results are also supported by observed data from several nations following their nationwide roll-outs of ChAdOx1 nCoV-19/AZD1222, but, they do imply that efficacy, even against severe infection, deteriorates with time [30,83]. These effectiveness statistics are in line with data from immunogenicity trials, which showed that vaccination recipients had strong binding and neutralizing antibody responses [12,84,85]. In some people who have received vaccinations, the delta and omicron variants elude immunological responses [1,2,3,4,53,55](https://www.who.int/publications/m/item/draft-landscape-of-COVID-19-candidate-vaccines) (accessed on 7 August 2022).

The phase I/II clinical trial conducted on adults aged 18–55 followed the induction of humoral and cellular immune responses after a single dose of ChAdOx1. The results showed that CD^4+^ T cells produced IFN- and TNF-, which induced the Th1 response. The antibodies produced were predominantly IgG1 and IgG3 classes. In addition, CD^8+^ T cells of monofunctional, polyfunctional and cytotoxic natures were activated [86]. Another study showed the induction of a stronger humoral and cellular immune response in people who received theChAdOx1 first vaccine dose of ChAdOx1 and a booster dose of BNT162b2, also referred to as the heterologous vaccination, as compared to the patients receiving both doses of the homologous vaccine [87].

The side effects include both local and systemic adverse reactions such as pain, swelling, itching, diarrhea, joint pain, and chills [87].

### 4.6. Ad5-Derived Vaccine (CanSino Biologics) 

This vaccine is built on an adenovirus 5 vector that produces the spike protein but is replication-incompetent. It is administered intramuscularly as a single dose. Pre-existing immunity to adenovirus 5 and older age was linked in early clinical studies to lower titers of binding and neutralizing antibodies after vaccination; this may restrict its applicability in areas where pre-existing immunity is common [10]. A randomized phase III study found that the vaccination was effective in preventing symptoms of infection in 57.5% (95% CI 39.7–70.0) of cases and in preventing severe illness in 91.7% (95% CI 36.1–99.0) of patients [88]. This vaccination is offered in China and several other nations, such as Pakistan and Mexico (1–4, https://www.who.int/publications/m/item/draft-landscape-of-COVID-19-candidate-vaccines) (accessed on 7 August 2022). 

In a phase II clinical trial conducted on 604 volunteers, neutralizing antibodies were detected in 95% of individuals and the production of Th1-specific IFN-γ was observed in 90% of individuals [10]. In a recent Phase 4 trial, the heterologous booster dose of Ad5-nCov or Convidecia in people with two prior doses of CoronaVac elicited better neutralizing antibody titers compared to the homologous booster doses of CoronaVac [89].

The most common side effects include local reactions such as pain (54% of vaccine recipients) and systemic reactions include fever (46% of vaccine recipients), fatigue (44% of vaccine recipients), headache (39% of vaccine recipients) and muscle pain (17% of vaccine recipients) [10].

### 4.7. Gam-COVID-Vac/Sputnik V (Gamaleya Institute) 

Two replication-deficient adenovirus vectors are employed to produce a full-length spike glycoprotein in a vaccine candidate developed by Gamaleya Institute. Adenovirus 26 vector is the first dose of the vaccine administered intramuscularly, and an adenovirus 5 vector boosting dose is administered 21–23 months later (https://sputnikvaccine.com/newsroom/pressreleases), (accessed on 19 September 2022). This vaccination is accessible in Mexico as well as in a number of other nations, including Russia. At the time of the second dose, this vaccine had 91.6% (95% CI 85.6–95.2) effectiveness in avoiding symptomatic COVID-19, according to an interim analysis of a phase III trial [15]. The placebo group contained all 20 cases of severe COVID-19 that manifested 21 days following the initial treatment. Local and systemic flu-like symptoms occurred 15% and 5% more frequently in the vaccination group, respectively. No major side effects were linked to the vaccination (1–4, https://www.who.int/publications/m/item/draft-landscape-of-COVID-19-candidate-vaccines) (accessed on 7 August 2022). 

A longitudinal study conducted on 118 volunteers showed the stable sustenance of neutralizing antibodies against the VOCs (α, β, γ & δ) and a local VOI over 6 months. Additionally, the cross-neutralizing activity shows a significant reduction in the escape of these VOCs to the neutralizing antibodies (89). In a recent study conducted on non-human primates, the intranasal delivery of Sputnik V induced a strong local and systemic immune response. The production of marked levels of neutralizing antibodies in the serum, proliferation of antigen-specific CD^4+^ & CD^8+^ T cells and a proinflammatory cytokine response protected against the severe lung pathophysiology induced by SARS-CoV-2 which may lead to sterilizing immune response [90].

The side effects include injection site pain, fatigue, headache, body ache, fever, drowsiness, and chills [91].

### 4.8. WIV04 and HB02 (Sinopharm) 

These inactivated, whole-virus vaccines, each containing an adjuvant like aluminium hydroxide, are developed using two COVID-19 isolates obtained from China. An alternative name for HB02 is BBIBP-CorV. Each of them is administered intramuscularly twice, 28 days apart. In a phase III efficacy trial, vaccination effectiveness for WIV04 and HB02, when compared to an alum-only placebo, was assessed to be 73% (95% CI 58–82) and 78% (95% CI 65–86), respectively [92]. There were just two severe instances, both in the placebo group. Similar frequencies of systemic and injection site responses were seen in all three groups (eg, pain in 20–27%, headache in 13%, fatigue in 11%). China and a few other nations, such as Hungary and the United Arab Emirates, have access to these vaccinations (1–4, https://www.who.int/publications/m/item/draft-landscape-of-COVID-19-candidate-vaccines) (accessed on 7 August 2022).

In a recent cohort-based study conducted in Pakistan, the titers of neutralizing antibodies after two doses of the BBIBP vaccine were lower than those induced by the natural SARS-CoV-2 infection. However, vaccine administration in people with prior exposure to SARS-CoV-2 significantly increases the neutralizing antibody titers [93]. In another study conducted in Thailand, the efficacy of heterologous booster doses of mRNA (BNT162b2 or mRNA-1273) or recombinant adenovirus-based vaccine (AZD122) on BBIBP-CorV-primed people was assessed. When patients who had received two doses of BBIBP-CorV were given a heterologous booster dose, the resulting neutralizing antibodies inhibited the delta and omicron variants by 90% and 70%, respectively, in addition to stronger IFN- production of antigen-specific CD^4+^ T cells [94].

The common side effects of the Sinopharm vaccine after the first dose are pain at the injection site, headache, and fatigue. The adverse reactions after the second dose are lethargy, fatigue, and tenderness in both males and females [95].

### 4.9. CoronaVac (Sinovac)

This inactivated COVID-19 vaccine was created in China and contained an aluminium hydroxide adjuvant. Two intramuscular doses of the vaccine are administered, separated by 28 days. The effectiveness of the vaccination was 83.5% (95% CI 65.4–92.1) according to preliminary findings of a phase III study conducted in Turkey [96]; however, smaller trials from other nations have also indicated higher efficacy rates [97,98]. A study in Brazil found lower vaccine effectiveness among adults older than 70 years in the context of the prevalent Gamma variant (47, 56, and 61 percent against COVID-19, hospitalization, and death, respectively [99]. The observational survey in Chile, which included over 10 million participants, estimated vaccine effectiveness to be 70% for trying to prevent COVID-19 and 86–88% for preventing hospitalization or death [100]. China and a few other nations, such as Chile, Mexico, Brazil, Indonesia, and Turkey, have access to this vaccine [1,2,3,4].

The vaccine induced neutralizing antibodies and virus-specific T cells in a gradual manner, reaching a peak after the four-week booster vaccine dose. These T cells showed the ability to neutralize the VOCs delta and omicron variants in phase III clinical trials [101,102].

The side effects of CoronaVac are similar to the Sinopharm vaccine and include muscle pain, diarrhea, and fatigue subsiding within 48 h [103].

### 4.10. Covaxin (Bharat Biotech/Indian Council of Medical Research)

Covaxin is a virus-inactivated vaccine (also known as BBV152) developed jointly by Bharat Biotech and ICMR in India. It contains aluminium hydroxide and a toll-like receptor agonist adjuvant. It was created and is now being utilized in India. Two intramuscular injections are administered, separated by 29 days. In a randomized experiment, the vaccination’s effectiveness against COVID-19 symptoms was 78% (95% confidence interval [CI] 65–86); one instance of severe COVID-19 infection occurred in the vaccinated group in comparison to 15 cases in the placebo group [104]. With the exception of one case of immune thrombocytopenic purpura, no serious side effects were linked to the immunization [1,2,3,4].

In a recent study, the persistence of humoral and cellular immune responses was followed up to six months post-vaccination. The antigen-specific CD^4+^ T cells producing numerous cytokines were detected in 85% of individuals, while the CD^8+^ T cells were detected in 50% of individuals. It was also found that the central memory T cells stayed around for up to six months after a vaccination [105].

The phase I and phase-II clinical trials presented side effects such as nausea, chills, body ache, and fatigue with no severe adverse reactions [104].

### 4.11. ZyCoV-D

The first DNA COVID-19 vaccine to be made accessible was authorized in August of 2021 in India, developed by Zydus Cadila [106]. A high-pressure stream from a needleless device administers the vaccination subcutaneously. In a study with 28,000 people aged 12 or older, the vaccine’s effectiveness against symptomatic COVID-19 was 67% (95% CI 47.6–80.7) after three doses spaced 28 days apart. In the placebo group, there was just one severe incidence of COVID-19 [1,2,3,4,107].

After the first dose of ZyCoV-D, there were a few minor side effects in the Phase 3 clinical trials, but the next doses went well [107].

## 5. Evolution of SARS-CoV-2-Alpha, Beta, Delta, Omicron, Their Discovery, Infectivity

The recently found variants of SARS-CoV-2 have been classified as either the Variants of Concern (VOCs), which include the Alpha, Beta, Gamma, and Delta variants, or the Variants of Interest (VOIs), which include the Eta, Iota, Zeta, and Epsilon variants. The Omicron variant is also considered to be a VOC [108,109].

The transmission rate was greatest in the alpha variant case after rigorous analysis of the evolutionary mechanisms, regional spread and distribution pattern, transmission trends, and diversity of mutational events among all the emerging viral variants [110]. Numerous investigations claim that mutations in the S protein are the most concerning variations in the virus because the main goal of vaccinations is to induce antibodies against the S protein’s components [110,111]. As a matter of fact, five VOCs have been identified in numerous studies based on infection severity and transmission potential owing to several mutations or amino acid changes [109,110,112,113,114]. According to studies looking at the new COVID-19 variants, a higher rate of mutations in S protein was observed, which may lead to structural changes that reduce the vaccine’s effectiveness (80–82, 85–87).

The different variants of SARS-CoV-2 were detected in various locations, including the alpha variant (UK), beta variant (South Africa), gamma variant (Brazil), mu variant (Colombia), lambda variant (Peru), epsilon variant (California), which are termed the variants of concern (VOCs) [109,112,113,114]. As previously mentioned, the mutations may change how the receptor binding domain (RBD) within the S protein interacts with the human ACE2 receptor, thus circumventing the effect of employed vaccines and changing the pace of viral infection [114,115]. The host alterations may be facilitated by the altered interaction of the virus with ACE2, further increasing the viral transmissibility [114]. For instance, in February, following the start of the pandemic, a single amino acid variation (D614G) in the spike (S) protein was discovered, enhancing the transmission ability of the mutated virus in Europe [110,113,115]. By May 2020, another amino acid variation, G614, displayed increased transmissibility by 70% [115]. Therefore, the efficacy of current vaccines highly depends upon the ongoing mutation in viral S protein [4,112,113,116,117].

### 5.1. Alpha and Beta Variants

The Alpha variant from the UK displayed about 14 mutations in the RBD site, with a few of them causing the deletion of three amino acids, thus increasing the transmission ability of the virus by 50%. The beta variant from South Africa and gamma variant from Brazil and the UK harbor two extra mutations leading to significant changes within the S protein. These mutations appear to make the virus more transmissible. In the virus’s infection and transmission ability, three mutations, specifically P681H, N501Y, and H69-V70 del observed in the alpha variant, are fascinating [114]. Additionally, the N501Y mutation in the RBD domain is seen in both the Alpha and Beta forms, increasing its affinity for the human ACE2. It also enhances the ability of the virus to get transmitted and escape from the host defense mechanisms [114].

### 5.2. Gamma Variant

The Gamma variant was mainly found in Brazil in 2020, having 12 variations in the S protein, with 3 of them located in the RBD region. This variant is 1.7–2.4 times more contagious and 1.2–1.9 times deadlier [118,119].

### 5.3. Delta Variant

The Delta variant (VUI-202012/01) was first detected among Indian travelers returning from Tanzania and South Africa (85). The Indian counterpart of the Delta variant possesses two point mutations, Leu452Arg and Glu484Gln. Of these two mutations, Leu452Arg facilitates the ability of the virus to connect with ACE-2 receptors in the host through its spike protein. L452R promotes viral proliferation and can elude antibodies [109]. It should be noted that mutations within the RBD region (L452R and E484Q) and the furin cleavage site (P681R) may have caused the increase in ACE2 binding and also enhanced the rate of S1-S2 cleavage, which would have improved transmissibility (86).

### 5.4. Omicron Variant

The first occurrence of the Omicron variant was verified in November 2021 in South Africa and Botswana [120,121,122,123,124]. The latest Omicron variant shows over 50 mutations, half located in the S protein’s RBD region [109,124]. Only a handful of these mutations had previously been found [124]. The Omicron variant features a deletion in the spike protein (position 69–70), similarly to the Alpha variant; three important mutations that provide immunological escape are found in the Beta and Gamma variants [124]. Additionally, the Centers for Disease Control (CDC) discovered at least 15 essential changes in the S protein that may have improved the viral ability to infect, particularly when compared to the lethal Delta variant [121,124]. This variant is more dangerous because of the several mutations at the furin cleavage site and RBD of the S protein, which have also been detected in the delta variant [109,114,124]

Recent studies have shown that single nucleotide variation exists in the SARS-CoV-2 viruses within the same host, termed the intrahost Single Nucleotide Variations (iSNVs). These variations emerge randomly and may or may not get fixed within the host. If some of these variations are fixed, they get transmitted within the population, and if they do not get fixed, they may lead to the generation of genetically diverse viral populations. The fixation of these variations may be either random or stochastic [125] or involve deterministic components [126]. The genetic characteristics of these variations have been characterized in COVID-19 patients [127]. The iSNV analysis uses deep viral genome sequencing of the clinical samples obtained from COVID-19 patients. The non-synonymous variations are commonly observed in the iSNV pool but are less common at the single nucleotide polymorphism (SNP) level. This indicates the selection of a few mutations (negative selection) from a pool of iSNVs generated (positive selection) within the hosts [128]. The mutations associated with all the existing SARS-CoV-2 variants are highlighted in Table 3. 

## 6. Effect of Current Vaccines on COVID-19 Variants

The ChAdOx1-S/AZD1222 vaccine, the mRNA-1273 vaccine, the mRNA-BNT162b2 vaccine, the JNJ-78436735 vaccine, and the NVX-CoV2373 vaccine are now the most popularly marketed vaccines [3,4,129]. BNT162b2 and mRNA-1273 were shown to induce neutralizing antibodies with reduced efficiency against the Alpha variant. In contrast, NVX-CoV2373 was established to impart 85.6% effectiveness in the UK population, whereas 60% efficacy was observed in the South African population [108,111,130]. Ad26.COV2.S vaccine displayed 64% efficacy among the Brazilian inhabitants, and 52% efficiency among the South African inhabitants against B.1.351 variants (N501Y.V2 lineage, made up of three receptor-binding domain mutations and five additional N-terminal domain mutations), and NVX-CoV2373 indicated 49% efficacy in the South African population [108]. The P.1 variation has been susceptible to the Pfizer/BioNTech BNT162b2 vaccine, even though this variant can evade inhibition by producing neutralizing antibodies [117]. The vaccines from Pfizer-BioNTech, Moderna, Oxford/AstraZeneca, and Sinovac are 85%, 78%, less than 70%, and 66% effective against gamma and delta forms, respectively [131]. The BNT162b2 mRNA vaccine has displayed the ability to cross neutralize a few of the circulating Delta variants, while the effectiveness of Ad26.COV2.S against this variant lowered from 66.9 to 60% after two doses [110]. While the Pfizer and AstraZeneca vaccine candidates displayed less efficiency in eliminating this variant compared to the Alpha variant, the Moderna vaccine showed an efficacy of about 94.1% against the Delta variant compared to BioNTech and the Johnson and Johnson vaccines [110,132]. The Oxford/Astra Zeneca vaccine candidate was nine times less effective than the RNA vaccine. However, the Biontech/Pfizer and Moderna mRNA vaccines were able to lower the infectivity of the Alpha versions [4,133]. The beta variants demonstrated the vaccine immunization escape technique employing convalescent plasmas in multiple instances [4,134]. A phase-III trial of Ad26.COV2.S with 40,000 participants showed that it worked almost 66.9% of the time [114].

Based on the experimental observation, it can be speculated that the variant of concern may eventually resist the neutralizing effect of antibodies induced by vaccines currently in use [114]. For instance, in the case of the Moderna or Pfizer vaccines, a 2.7–3.8-fold reduction was observed in the Alpha variant [114,121,133]. The Beta variant was more resistant to vaccination than the Alpha variant [121,135]. The Pfizer vaccine’s effectiveness against the Delta variant dropped from 94 to 64% [110]. In South Africa, the vaccine effectiveness against the omicron variant was noted to be 70%, while it was nearly 93% against hospitalized COVID-19 patients exposed to the Delta variant [122]. This demonstrated the decreased vaccine protection against the Omicron variants. The provision of a booster dose of vaccine can supplement this loss of vaccine efficacy [123]. More so than all other current VOCs and VOIs, the massive number of mutations inside the Omicron variant led to a considerable decrease in the neutralization of viral S protein using human convalescent sera from patients infected with COVID-19 [123]. This variation was notable for exhibiting an enhanced propensity for host immunological escape, which is concerning given the current Omicron infection [121]. Even though clinical trials are still being conducted, the current vaccines’ effectiveness is widely acknowledged worldwide. Vaccines were developed to lessen the severity with which disease symptoms develop and to decrease the rate of hospitalization. As a result, booster doses are necessary that could extend the duration of the neutralizing antibodies in hosts. In 87 people between 1.3 and 6.2 months post-COVID-19 infection, the humoral memory response evaluation displayed that IgM and IgG titers against the RBD decreased over time [136]. The memory B cells specific for RBD were shown to have an unexpected property of clonal turnover, somatic hypermutation, resistance to changes in RBD, and an enhanced potency 6.2 months after infection [136,137,138]. Optimizing vaccination schedules and boosters as well as considering the effects of other protective measures such as wearing masks, social distancing, etc., can help sustain such efficacy [137,139]. Additionally, while the Omicron form may escape the immune protection induced by double vaccine doses, it can be circumvented by the booster vaccine doses [92].

## 7. Need for a Broad-Spectrum Vaccine against COVID-19 Variants

Given the rapid emergence of COVID-19 variants and the limitations of existing therapeutic strategies to control their spread, there is a strong need to develop broad-spectrum vaccine formulations or a panvaccine that may protect against all the existing and upcoming variants of the pathogen. For example, an ideal broad-spectrum therapeutic strategy should be equally effective against all or most existing variants of SARS-CoV-2. In addition, it should be able to induce both humoral as well as cell-mediated components of the adaptive immune response. Finally, it should also lead to the generation of memory responses against these variants. In this context, various researchers have recently shifted their focus to developing such multitargeted therapeutic strategies as discussed below.

### 7.1. Nanoparticle-Based Vaccine Formulations

The principle behind this technique was to build a nanocage consisting of engineered proteins that provide a tagging site for the viral proteins in the form of surface appendages. These nanocages may be modified to display proteins from only one or multiple viruses and may be termed homotypic or mosaic nanoparticles. Upon administration into the host, these engineered nanocages show viral antigenic fragments to the immune system and elicit the production of specific humoral and cell-mediated adaptive immune responses [140]. For example, in a recent study, about 8 different coronaviruses were chosen that have either caused a pandemic or have the potential to do so. The spike protein’s receptor-binding domain is made up of small pieces of protein that were put together on a nanoparticle scaffold [140].

In another study, the spike protein of COVID-19 was encapsulated within a ferritin nanoscaffold and liposomes. Ferritin monomers undergo self-assembly to generate scaffold systems that have been used as adjuvant or drug delivery systems [141]. When introduced in primates, this new vaccine, termed Spike Protein Feritin Nanoparticles Vaccine (SpFN), induced both virus-specific B and T cells. The serum obtained from vaccinated animals showed high titers of neutralizing antibodies effective against various COVID-19 variants. Two doses of SpFN (50 ug) within a 28-day interval between them induced a TH1 response and the generation of neutralizing antibodies against the wild-type viral strain and its variants. The induction of humoral and cell-mediated immune responses inhibited virus replication in the upper and lower respiratory tracts in non-human primates [142].

### 7.2. Antibody-Based Vaccine Formulations

The neutralizing antibodies isolated from the convalescent sera of COVID-19 patients have been recently shown to neutralize many of the existing variants of SARS-CoV-2, thus being termed the Broadly Neutralizing Antibodies (bNAbs).

Recently, a combination of 30 antibodies was characterized to offer protection against all the variants of SARS-CoV-2 and the other coronavirus types found in other animals such as bats and pangolins. The antibodies were isolated from 107 COVID-19 patients who had developed hybrid immunity and showed a significant ability to bind to the spike proteins of both SARS-CoV-1 and 2. These antibodies targeted the conserved protein segments common to all the coronaviruses. When mice were given these antibodies and then infected with SARS-CoV-1 and 2, they had less virus in their lungs than mice that had not been given these antibodies [143].

In one of the studies, the convalescent sera collected post-vaccination with the Ad5-nCoV vaccine was used to obtain bNAb against the SARS-CoV-2 variants. A monoclonal antibody termed ZWD12 exhibited efficacy against the Alpha, Beta, Gamma, Kappa, Delta, and Omicron variants through the blockage of the binding of the spike protein with the ACE2 receptor. This mAb provided complete protection against all the variants of SARS-CoV-2 in a transgenic mouse model [144]. In another study, 1737 mAbs were purified from the convalescent sera of a 17-year-old COVID-19 patient [145]. From this pool of mAbs, a mAb termed DH1047 showed broad neutralization activity against not only the SARS-CoV-2 but also the other pre-emerging bat coronaviruses and their variants in mice [146]. A mAb known as SP1–77 was obtained from a mouse model in which the B cell repertoire is generated via V(D)J recombination between a human light and heavy chain. This antibody neutralizes all the known SARS-CoV-2 variants through the inhibition of membrane fusion [147].

### 7.3. mRNA-Based Vaccine Formulations

Recently, a broad-spectrum mRNA vaccine, RQ3013, was developed to protect against the variants of concern. This vaccine consists of mRNAs modified by the incorporation of pseudouridine and encapsulated in liposomes—these mRNAs code for viral spike proteins that harbour all the mutations detected in VOCs. The vaccine has shown to induce immune responses in various animal models, including primates, hamsters and mice, with high antibody titers that can neutralize the wild type and the α, β, γ, δ and omicron variants of COVID-19. Two doses of the mRNA vaccine showed protection of the respiratory tract from getting infected by the variants as mentioned above. In addition, the vaccine formulation was found to be safe and well tolerated in these animal models [148].

### 7.4. CRISPR Based Vaccine Formulations

CRISPR-Cas13d has shown broad-spectrum inhibition activity against various COVID-19 variants. This inhibition depends upon the cRNA co-localization with Cas13d and the target RNA of the COVID-19 variant. Cas13d can also enhance the anti-viral activity of small-molecule inhibitors. Using liposome-based RNA delivery, Cas13d can inhibit the COVID-19 variants in human airway epithelium cells. This strategy can work well with both the vaccines and the drugs that fight viruses [149].

### 7.5. Circular RNA-Based Vaccine Formulations

Recently, a circular RNA-based vaccine induced the production of neutralizing antibodies as well as virus-specific T cells. The circular RNA encodes for the RBD region of the virus spike protein and showed robust protection upon administration in rhesus monkeys and mice. The vaccine sustained antigen production, provided higher and longer-term protection against delta and omicron variants, and could also boost the effects of other vaccines [150].

### 7.6. siRNA-Based Vaccine Formulations

siRNAs show great potential in constructing a broad-spectrum vaccine formulation, as they target mRNA and can be artificially modified to target multiple viruses simultaneously. It consists of dsRNA, 20 nucleotides long, which, on entering the host cytosol, modulates the expression of the target gene depending on the sequence complementarity with mRNA [151]. The delivery of naked siRNA into pulmonary cells has been tried previously through the inhalation route in mice [152,153,154]. In 2010, it was extended to inhibit syncytial virus replication through the intranasal administration of naked siRNA (ALN-RSV01) through the spray. The treatment showed a significant reduction in RSV prevalence in clinical trials (122) and reduced the risk of developing pulmonary complications post-infection in patients with lung transplants [155].

These results point out that such a siRNA delivery system could also be applied against all the variants of COVID-19 by constructing siRNA that could target a region conserved in all the variants. Recently, a modified siRNA preparation C6G25S was administered using the aerosol mode to inhibit SARS-CoV-2 variants effectively. This vaccine inhibited all the variants at picomolar concentrations and prevented generating and releasing viral progeny in the lungs. Moreover, it could decrease the viral load by 96% with a concomitant decrease in the virus-induced pulmonary damage, thus providing a practical approach to combat the SARS-CoV-2 variants [156].

**Figure 2:**
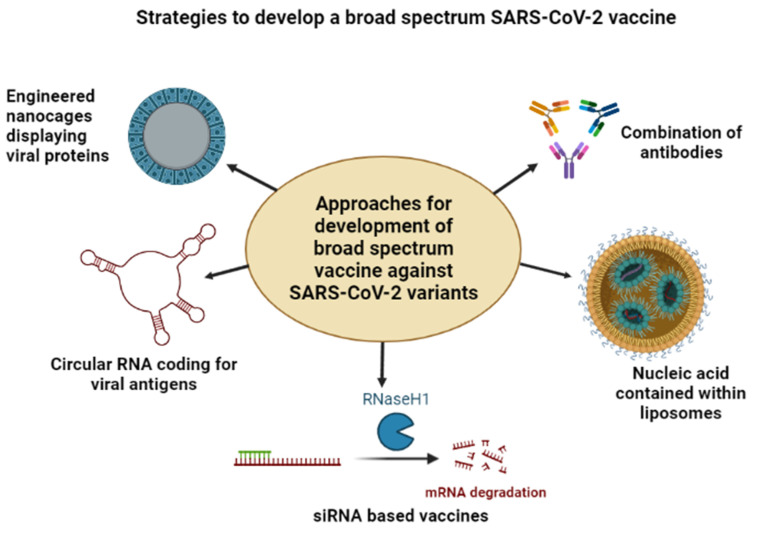
Strategies illustrating the methodology for the development of broad-spectrum SARS-CoV2 vaccine.

The recent approaches aimed at developing a broad-spectrum vaccine against the existing SARS-CoV-2 variants have been summarized in Figure 2.

## 8. Conclusions

This review gives detailed insights about the evolution of SARS-CoV-2 leading to the generation of several variants, the efficacy of existing preventive strategies against these variants, and the need for a broad-spectrum therapeutic approach. The existing treatment regime has found limited success in controlling the spread of these variants. Therefore, there is a growing need for creating a broad-spectrum therapeutic approach that could inhibit all the existing and future variants of SARS-CoV-2. We have covered all the latest discoveries that attempt to generate such broad-spectrum vaccines.

## Figures and Tables

**Table 1:** Countries with a maximum number of COVID-19 induced deaths (above 0.1 million).

Sr. No.	Country	Total COVID-19 Induced Deaths
**1.**	United States of America	1,020,405
**2.**	Brazil	678,715
**3.**	India	526,477
**4.**	Russian Federation	382,560
**5.**	Mexico	327,750
**6.**	Peru	214,364
**7.**	United Kingdom	183,953
**8.**	Italy	172,397
**9.**	Indonesia	157,028
**10.**	France	148,833
**11.**	Germany	144,360
**12.**	Iran	142,134
**13.**	Colombia	140,845
**14.**	Argentina	129,369
**15.**	Poland	116,608
**16.**	Spain	110,713
**17.**	Ukraine	108,713
**18.**	South Africa	101,982

**Table 2:** Data on COVID-19 vaccines administered (by country) as of 7 August 2022.

Sr. No.	Country	No. of People with Double Dose of COVID-19 Vaccines
**1.**	India	935.52 million
**2.**	United States	223.04 million
**3.**	Brazil	170.17 million
**4.**	Mexico	92.33 million
**5.**	Russia	82.58 million
**6.**	Germany	64.74 million
**7.**	Turkey	57.89 million
**8.**	France	54.53 million
**9.**	United Kingdom	53.71 million
**10.**	Italy	50.82 million
**11.**	Spain	41.28 million
**12.**	Chile	18.04 million
**13.**	Israel	6.72 million
**14.**	Hungary	6.41 million
**15.**	Uruguay	3 million
**16.**	Bahrain	1.24 million

**Table 3:** Mutations associated with SARS-CoV-2 variants.

Sr. No.	Variant	Place and Year of Discovery	No. of Mutations in Viral Genome	Most Significant Genetic Mutations	Phenotypic Effect
**1.**	Alpha (B.1.1.7)	United Kingdom Sept. 2020	17	His69_Val70 deletion, Tyr144 deletion, Asn501Tyr, Ala570Asp, Asp614Gly, Pro681His, Thr716Ile, Ser982Ala, and Asp1118His	Enhanced affinity towards the ACE-2 receptors leading to increased viral adhesion and invasion of host cells
**2.**	Beta (B.1.351)	South Africa May 2020	8	Leu242_Leu244 deletion, Asp80Ala, Asp215Gly, Lys417Asn, Glu484Lys, Asn501Tyr, Asp614Gly, and Ala701Val	Enhanced binding affinity of S protein with hACE-2 receptors leading to higher transmission risk
**3.**	Gamma (P.1)	Brazil November 2020	12	Leu18Phe, Thr20Asn, Pro26Ser, Asp138Tyr, Arg190Ser, Lys417Thr, Gly484Lys, Asn501Tyr, Asp614Gly, His655Tyr, Thr1027Ile, and Val1176Phe	1.7- to 2.4-fold more transmissibility and 1.2 to 1.9 times in increased mortality rate
**4.**	Delta (B.1.617.2)	India October 2020	9	Glu156_Phe157 deletion, Thr19Arg, Gly142Asp, Arg158Gly, Leu452Arg, Thr478Lys, Asp614Gly, Pro681Arg, and Asp950Asn	Increased viral replication and transmission ability causing a higher infection rate in non-vaccinated people
**5.**	Omicron (B.1.1.529)	India and South Africa, November 2021	97 (34 mutations in BA.1 lineage, 35 mutations in BA.1.1 lineage, and 28 mutations in BA.2 lineage	Gly339Asp, Asn440Lys, Ser477Asn, Thr478Lys, Gln498Arg, Asn501Tyr, Lys417Asn, Gly446Ser, Glu484Ala, Gln493Arg, Gly496Ser, Gln498Arg, and Asn501Tyr	Increased the binding affinity to hACE-2 making it highly contagious but less severe
**7.**	Lambda (C.37)	Peru, August 2020	7	Gly75Val, Thr76Ile, Arg246_Gly252 deletion, Leu452Gln, Phe490Ser, Asp614Gly, and Thr859Asn	Enhanced transmissibility thus increasing prevalence and morbidity.

## Data Availability

Not applicable.
